# Functional remodelling of perinuclear mitochondria alters nucleoplasmic Ca^2+^ signalling in heart failure

**DOI:** 10.1098/rstb.2021.0320

**Published:** 2022-11-21

**Authors:** Julia Voglhuber, Michael Holzer, Snježana Radulović, Phung N. Thai, Natasa Djalinac, Ingrid Matzer, Markus Wallner, Heiko Bugger, Andreas Zirlik, Gerd Leitinger, Elena N. Dedkova, Donald M. Bers, Senka Ljubojevic-Holzer

**Affiliations:** ^1^ Department of Cardiology, Medical University of Graz, Graz, Austria; ^2^ BioTechMed-Graz, Graz, Austria; ^3^ Division of Pharmacology, Otto-Loewi Research Centre, Medical University of Graz, Graz, Austria; ^4^ Research Unit Electron Microscopic Techniques, Division of Cell Biology, Histology and Embryology, Gottfried Schatz Research Center, Medical University of Graz, Graz, Austria; ^5^ Department of Internal Medicine, Cardiovascular Medicine, University of California Davis, Davis, CA, USA; ^6^ Lewis Katz School of Medicine, Temple University, Cardiovascular Research Center, Philadelphia, PA, USA; ^7^ Department of Pharmacology, University of California Davis, Davis, CA, USA; ^8^ Department of Molecular Biosciences, University of California Davis, Davis, CA, USA; ^9^ Molecular Biology and Biochemistry, Gottfried Schatz Research Center, Medical University of Graz, Graz, Austria

**Keywords:** nuclear calcium, perinuclear mitochondria, transverse aortic constriction, remodelling, heart failure

## Abstract

Mitochondrial dysfunction in cardiomyocytes is a hallmark of heart failure development. Although initial studies recognized the importance of different mitochondrial subpopulations, there is a striking lack of direct comparison of intrafibrillar (IF) versus perinuclear (PN) mitochondria during the development of HF. Here, we use multiple approaches to examine the morphology and functional properties of IF versus PN mitochondria in pressure overload-induced cardiac remodelling in mice, and in non-failing and failing human cardiomyocytes. We demonstrate that PN mitochondria from failing cardiomyocytes are more susceptible to depolarization of mitochondrial membrane potential, reactive oxygen species generation and impairment in Ca^2+^ uptake compared with IF mitochondria at baseline and under physiological stress protocol. We also demonstrate, for the first time to our knowledge, that under normal conditions PN mitochondrial Ca^2+^ uptake shapes nucleoplasmic Ca^2+^ transients (CaTs) and limits nucleoplasmic Ca^2+^ loading. The loss of PN mitochondrial Ca^2+^ buffering capacity translates into increased nucleoplasmic CaTs and may explain disproportionate rise in nucleoplasmic [Ca^2+^] in failing cardiomyocytes at increased stimulation frequencies. Therefore, a previously unidentified benefit of restoring the mitochondrial Ca^2+^ uptake may be normalization of nuclear Ca^2+^ signalling and alleviation of altered excitation–transcription, which could be an important therapeutic approach to prevent adverse cardiac remodelling.

This article is part of the theme issue ‘The cardiomyocyte: new revelations on the interplay between architecture and function in growth, health, and disease’.

## 1. Introduction

Despite major improvements in available therapeutic options, heart failure (HF) remains one of the leading causes of death worldwide [1]. While common pharmacotherapeutics target systemic changes in the neurohormonal status of HF patients [2], no intervention that directly improves cardiomyocyte function and viability has been successfully implemented in clinical practice. Thus, elucidating molecular changes occurring at a cellular level during the initiation and progression of HF is critical for the development of new therapeutic strategies. Mitochondrial dysfunction in cardiomyocytes has been identified as one of the hallmarks of HF development, and it has been shown to contribute to impaired contractility of the heart [3] and survival of cardiomyocytes [4]. Remarkably, all mitochondrial functions affected in HF require tight regulation of calcium (Ca^2+^) fluxes [5] and include reduced cellular respiration rate [6–8], increased production of reactive oxygen species (ROS) [9], impairment of intraorganellar Ca^2+^ cycling [10,11] and lower rates of mitophagy [12,13], making mitochondrial Ca^2+^ homeostasis an important determinant of cardiac health.

In cardiomyocytes, a transient rise in the cytoplasmic Ca^2+^ concentration, [Ca^2+^], occurs during each heartbeat. The coordinated regulation of Ca^2+^ cycling under the conditions of different workload demand is achieved by the close physical proximity of the main Ca^2+^ cellular store, the sarcoplasmic reticulum (SR), and mitochondria distributed throughout the cell. At the junction between the SR and mitochondria, a space known as the subcellular microdomain, local Ca^2+^ fluxes of high magnitude lead to mitochondrial Ca^2+^ uptake [14,15]. Mitochondrial Ca^2+^ entry is operated by the coordinated action of voltage-dependent anion channels (VDACs), located on the outer mitochondrial membrane [16] and the mitochondrial Ca^2+^ uniporter (MCU), located on the inner membrane [17], and it is critical for meeting the energy demands of cardiomyocytes [7,17,18]. For example, increased pacing frequency and adrenergic stimulation result in increased mitochondrial [Ca^2+^], which enhances tricarboxylic acid cycle dehydrogenases, leads to faster NAD(P)H reduction and finally increases ATP generation by feeding the electron transport in the respiratory chain (reviewed in [19]).

However, the process of mitochondrial Ca^2+^ uptake is compromised in HF [16,17,20,21] and exacerbated further by the reduction in relative mitochondrial content in early [22] and late [23] cardiac remodelling, supporting the idea that it is causally implicated in the pathogenesis of HF. Indeed, recent work has demonstrated that interventions leading to enhanced mitochondrial Ca^2+^ uptake can have beneficial effects on the development of HF [21,24].

Cardiomyocytes contain multiple subpopulations of mitochondria: subsarcolemmal (SSL), intrafibrillar (IF) and perinuclear (PN), all of which show distinct characteristics. SSL mitochondria are packed in spaces just under the plasma membrane, IF mitochondria are organized in long parallel threads surrounding the contractile myofilaments, where they form structural and functional complexes with the SR, while dense clusters of smaller grain-like mitochondria surrounding the nuclei are observed in PN regions [25]. Unlike the SSL and IF mitochondrial subpopulations, which were the focus of numerous previous investigations, the PN mitochondria have scarcely been studied. Lu *et al*. recently demonstrated that in contrast to IF mitochondria, which are remarkably static, PN mitochondria are relatively mobile, appear to participate in fission/fusion dynamics and play a central role in mitochondrial genesis and turnover [26]. Mitochondrial fission is required to create new mitochondria and segregate damaged ones for mitophagy, while mitochondrial fusion results in elongated mitochondria and allows content mixing between two fusing organelles. Hence, PN mitochondria appear to play an important role in regulating the adaptation of the mitochondrial network to meet the metabolic needs of the cell. Furthermore, PN mitochondria show higher autofluorescence of mitochondrial NADH than IF mitochondria [25], possibly owing to lower mitochondrial respiration and/or a shift in metabolism towards glycolysis [27].

Accumulating knowledge on mitochondrial dysfunction in HF has been recently reviewed [28] and it comprises redox imbalance, ROS-induced ROS generation, impaired mitochondrial Ca^2+^ homeostasis, increased glycolysis, decreased fatty acid oxidation, and increased inflammation and rates of cell death via mPTP opening. While initial studies have investigated functional remodelling of mitochondrial subpopulations, there is a striking lack of direct comparison between IF versus PN mitochondria during cardiac remodelling and its progression to HF. Furthermore, the functional consequences of mitochondrial dysfunction on nuclear signalling, including Ca^2+^ cycling, are yet to be elucidated. Here, we use multiple approaches to examine the morphology and functional properties of IF versus PN mitochondria in pressure overload-induced cardiac remodelling and failure in mice (via trans-aortic constriction, TAC), and as a proof-of-principle for clinical relevance of our findings, we repeated a subset of experiments in non-failing and failing human cardiomyocytes. We have demonstrated that PN mitochondria from failing cardiomyocytes are more susceptible to changes in mitochondrial membrane potential (Δ*Ψ*_m_), ROS generation and impairment in Ca^2+^ uptake compared with IF mitochondria at baseline and under physiological stress. We have also shown, for the first time to our knowledge, that under normal conditions PN mitochondrial Ca^2+^ uptake shapes nucleoplasmic Ca^2+^ transients (CaTs) and limits nucleoplasmic Ca^2+^ loading. The loss of PN mitochondrial Ca^2+^ buffering capacity translates into increased nucleoplasmic CaTs and may help to explain the disproportionate rise in nucleoplasmic [Ca^2+^] in failing cardiomyocytes at increased stimulation frequencies. Therefore, normalization of mitochondrial Ca^2+^ regulation may be a novel therapeutic approach to restore altered Ca^2+^-mediated transcription and prevent adverse cardiac remodelling.

## 2. Material and methods

The data supporting findings of this study are available from the Dryad Digital Repository: https://doi.org/10.5061/dryad.pvmcvdnn9. Materials and methods are described in detail in the electronic supplementary material.

All procedures involving animals were carried out in accordance with the Federal Act on the Protection of Animals (Medical University of Graz) or the NIH Guide for the Care and Use of Laboratory Animals (UC Davis) and were approved by the Institutional Animal Care and Use Committee. Human hearts (from patients and organ donors whose hearts could not be used for transplantation) were acquired via collaboration with the Division of Cardiac Surgery (Medical University of Graz). The use of human samples was approved by the Ethical Committee of the Medical University of Graz, and all experimental procedures were carried out in accordance with the Declaration of Helsinki.

## 3. Results

### (a) Distribution and morphology of intrafibrillar and perinuclear mitochondria in pressure overload

**Figure 1.**
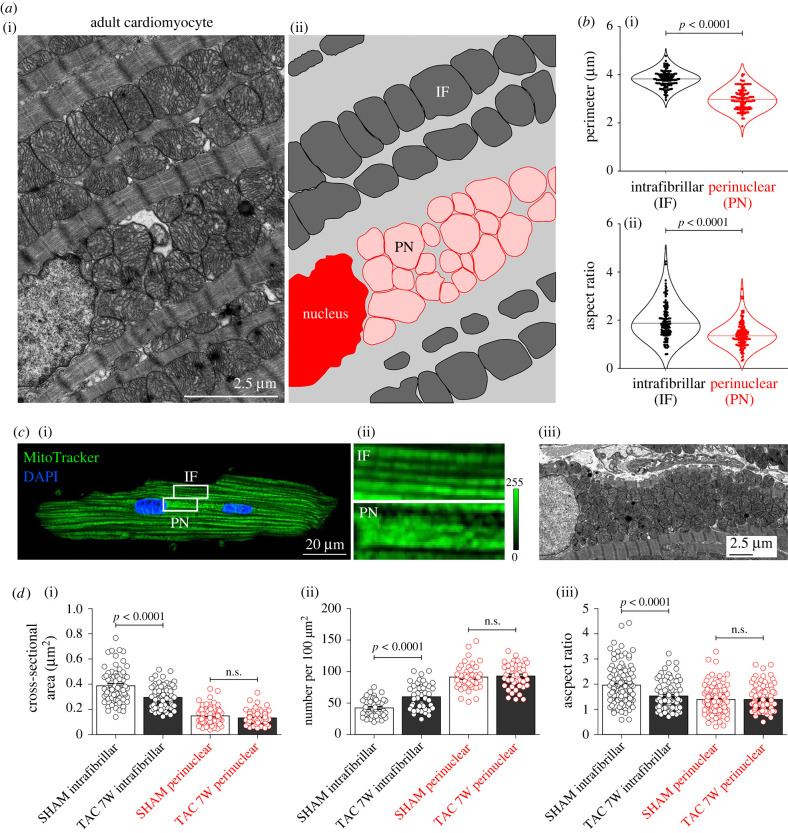
Intrafibrillar (IF) and perinuclear (PN) mitochondrial localization and their morphological alterations in failing mouse ventricular cardiomyocytes. (*a*) Representative EM images (i) and schematic overlay (ii) distinguishing populations of IF and PN mitochondria in left ventricular sections of a healthy mouse heart. (*b*) Average values of IF and PN mitochondrial perimeter (i) and aspect ratio (ii) in healthy mouse ventricular myocytes. *n* = 120 mitochondria from *N* = 3 mice per group. (*c*) Representative fluorescence image of a live adult mouse cardiomyocyte stained with MitoTracker® Green (i) and magnification of corresponding cell areas containing IF and PN mitochondria (ii). Nuclear localization was confirmed by DAPI staining. (iii) EM image of PN mitochondria accumulated in the space between two nuclei of a typically binucleated mouse ventricular myocyte. (*d*) Average values of IF and PN mitochondrial size (i), number per area (ii) and aspect ratio (iii) in ventricular cardiomyocytes isolated from sham- and seven-week post-TAC surgery mice. For pressure overload-induced changes in morphological parameters 68–120 individual mitochondria from *N* = 3 mice (4–8 cells) or 43–44 cellular sections (15 cells) per group were traced. *p*-values were calculated using Mann–Whitney test comparing TAC group to the respective sham control. n.s., not significant.

To address potential changes in the organization and function of mitochondrial subpopulations during pressure overload-induced HF, we first performed confocal and electron microscopy (EM) imaging of intact adult ventricular cardiomyocytes and mouse myocardium preparations, respectively. As previously observed in EM images of rabbit hearts [26], ultrastructural morphometric analysis showed that IF mitochondria are organized longitudinally, densely filling the space between the myofibrils, while PN mitochondria are tightly packed on the longitudinal poles of the nucleus (figure 1*a*), and in the space between the two nuclei in typically binucleated ventricular cells (figure 1*c*). Quantification of mitochondrial size and shape by individual tracing of mitochondria defined as organelles enclosed by a double contoured membrane in cytoplasmic and PN spaces showed that mitochondrial perimeter and aspect ratio are significantly different in the two subpopulations (figure 1*b*), with PN mitochondria being smaller and more spherical. Interestingly, IF mitochondria in TAC-operated mice at seven weeks post-intervention appeared smaller and rounder, and were increased in number per area when compared with controls (figure 1*d*), while these parameters remained unchanged for PN mitochondria. These data suggest a shift of typical IF mitochondria towards a phenotype more resembling that of PN mitochondria during the development of HF. As PN mitochondria are critically involved in mitochondrial genesis and turnover, this phenotypic shift could be a result of premature mitochondrial recruitment and/or delayed degradation with important functional consequences for cardiomyocyte bioenergetics.

### (b) Abundance of intrafibrillar and perinuclear mitochondria in hypertrophic and failing mouse ventricular cardiomyocytes

**Figure 2.**
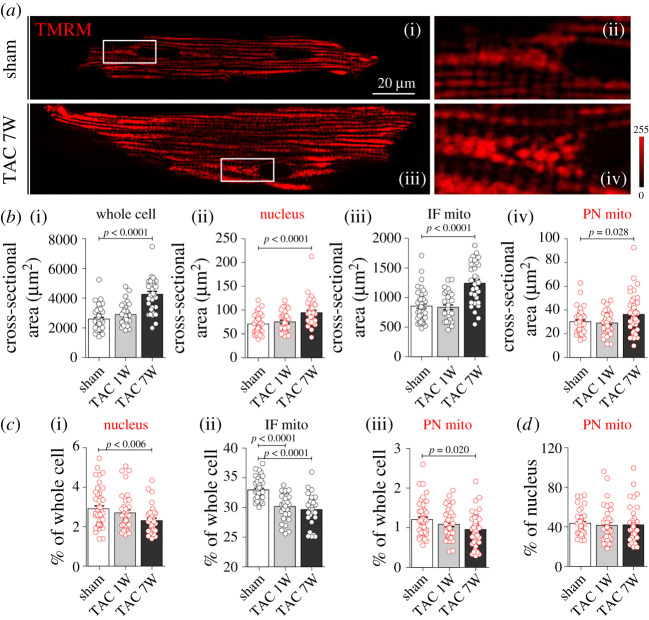
Abundance of intrafibrillar (IF) and perinuclear (PN) mitochondria in hypertrophic and failing mouse ventricular cardiomyocytes. (*a*) Representative fluorescence images of a live ventricular cardiomyocyte isolated from control and failing mice and stained with TMRM (i,iii), and magnification of corresponding cell areas containing PN mitochondria (mito.; (ii,iv)). (*b*) Average cross-sectional area of whole cell, nucleus, IF and PN mitochondria in ventricular cardiomyocytes isolated from sham- and TAC-operated mice one (1W) or seven (7W) weeks after the intervention. *p*-values were calculated using ANOVA with Dunnett *post hoc* test (with sham as control). (*c*) Average size of nucleus (i), IF (ii) and PN (iii) mitochondria relative to the corresponding size of cardiomyocyte, and (*d*) average size of PN mitochondria relative to the corresponding nuclei. *p*-values were calculated using Kruskal–Wallis with Dunn *post hoc* test comparing TAC 1W and TAC 7W with the respective sham control. (*b–d*) *n* = 30–46 cells isolated from *N* = 3–4 mice per group.

To assess the effect of short- and long-term pressure overload on the relative abundance of IF and PN mitochondria, we systematically quantified the subcellular composition of cardiomyocytes isolated from sham and one- and seven-week post-TAC hearts (figure 2). At the (sub)cellular level, cardiomyocyte and nuclear cross-sectional area and the area occupied by IF and PN mitochondria increased progressively in response to mechanical overload (figure 2*a,b*). However, myocyte growth was even greater, resulting in a reduced mitochondrial volume as a fraction of cell volume (figure 2*c*). This reduced relative mitochondrial volume was already decreased substantially for IF mitochondria at one week post-TAC, with similar values obtained at seven weeks post-TAC. These values are in remarkable agreement with a previous study using the same model, in which an advanced three-dimensional stereology method for quantification of cardiomyocyte composition was used [23]. The relative abundance of PN mitochondria within the cell tended to be lower at one week post-TAC and was statistically lower compared with sham at seven weeks post-TAC, although it remained proportional to the nuclear surface area (figure 2*d*). This relative decrease in IF and PN mitochondrial availability with increased heart size indicates that, despite the absolute increase in mitochondrial content, the mitochondria were diluted with respect to the growing myofibrillar elements.

### (c) Mitochondrial membrane potential (Δ*Ψ*_m_) of intrafibrillar versus perinuclear mitochondria in failing mouse and human cardiomyocytes

**Figure 3.**
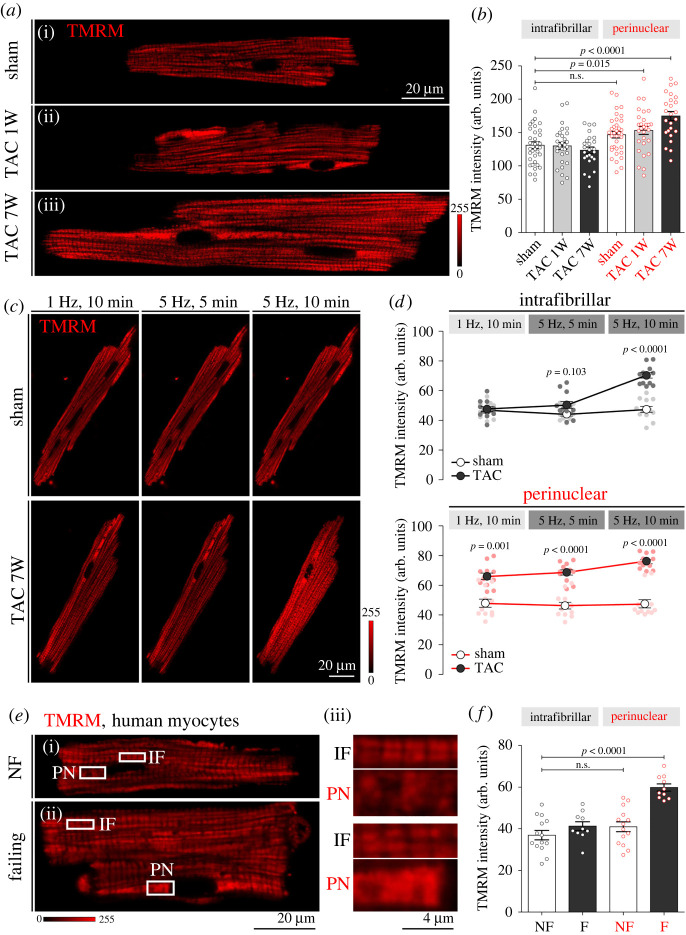
Mitochondrial membrane potential (Δ*Ψ*_m_) of intrafibrillar (IF) versus perinuclear (PN) mitochondria in control and failing mouse and human cardiomyocytes. (*a*) Original confocal images of cardiomyocytes from sham (i) and TAC (one (ii) and seven (iii) weeks after intervention) mice stained with TMRM. (*b*) Corresponding average values of TMRM fluorescence signal from IF and PN mitochondria. *n* = 25–35 cells isolated from *N* = 3 or 4 mice per group. *p*-values were calculated using ANOVA with Dunnett's *post hoc* test with IF sham as control. (*c*) Representative images of Δ*Ψ*_m_ in response to 5 or 10 min of high-frequency stimulation (5 Hz) in cardiomyocytes isolated from sham and seven-week post-TAC mouse hearts, detected by TMRM staining. (*d*) Corresponding average values of TMRM fluorescence signal from IF and PN mitochondria. *n* = 12 cells from *N* = 3 mice per group. *p*-values were calculated using Sidak's multiple comparisons test, following significant two-way repeated-measures ANOVA. (*e*) Original confocal images of cardiomyocytes from non-failing (NF, (i)) and failing (ii) human cardiomyocytes and magnification of corresponding cell areas containing IF and PN mitochondria (iii) stained with TMRM. (*f*) Corresponding average values of TMRM fluorescence signal from IF and PN mitochondria. *n* = 10–14 cells isolated from *N* = 3 hearts per group. *p*-values were calculated using ANOVA with Dunnett's *post hoc* test with IF NF as control. n.s., not significant.

Given that acute pressure overload reduces IF and PN mitochondrial cellular abundance in mice, we next sought to address the potential functional alterations of the two subpopulations over the course of TAC-induced remodelling. We found that mitochondrial membrane potentials (Δ*Ψ*_m_) determined using either potential-sensitive dye TMRM redistribution (electronic supplementary material, figure S1) or quench/de-quench modes (figure 3; please note that in this imaging mode increased fluorescence intensity implies depolarization of Δ*Ψ*_m_) were remarkably preserved in one- and seven-week post-TAC cardiomyocytes for IF mitochondria, while PN mitochondria exhibited a significant loss in Δ*Ψ*_m_, both at one and seven weeks post-TAC (figure 3*a*,*b*). Application of high-frequency pacing (5 Hz) had no effect on IF and PN Δ*Ψ*_m_ in cardiomyocytes isolated from sham controls, but pacing led to a significant decrease in Δ*Ψ*_m_ of IF mitochondria from late TAC mice, which matched the Δ*Ψ*_m_ levels of already depolarized PN mitochondria after 10 min of pacing (figure 3*c*,*d*). Similar experiments in cardiomyocytes isolated from human control and failing myocardium confirmed the inability of PN mitochondria to maintain Δ*Ψ*_m_ in HF (figure 3*e*,*f*), underscoring the clinical relevance of the data obtained in the experimental mouse model.

### (d) Reactive oxygen species production and Ca^2+^ uptake of intrafibrillar versus perinuclear mitochondria in control and failing mouse cardiomyocytes

**Figure 4.**
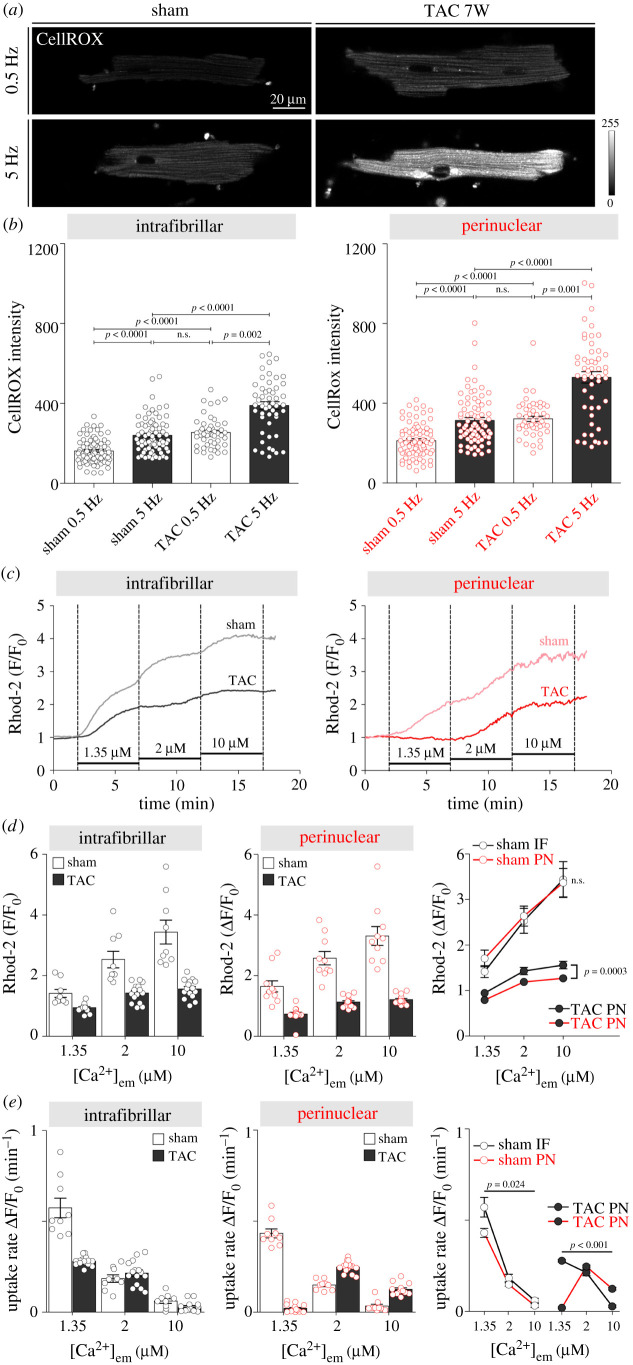
ROS production and Ca^2+^ uptake of intrafibrillar (IF) versus perinuclear (PN) mitochondria in control and failing mouse cardiomyocytes. (*a*) Representative images of ROS production in response to 10 min of high-frequency stimulation (5 Hz) in cardiomyocytes isolated from sham and seven-week-old TAC mouse hearts, detected by CellROX® Deep Red reagent staining. (*b*) Corresponding average values of CellROX® fluorescence signal from IF and PN mitochondria. *n* = 60–80 cells from *N* = 3 mice per group. *p*-values were calculated using Kruskal–Wallis with Dunn's *post hoc* test. (*c*) Representative tracings of IF and PN mitochondrial Ca^2+^ uptake in permeabilized cardiomyocytes isolated from sham and eight-week post-TAC mouse hearts using the calcium indicator Rhod-2 after the addition of 1.35, 2 and 10 µM Ca^2+^. Mean group data showing (*d*) mitochondrial Ca^2+^ uptake amplitude and (*e*) uptake rate in the four groups. *n* = 9–15 cells from *N* = 3 mice per group. *p*-values were calculated using Sidak's multiple comparisons test, following significant two-way repeated-measures ANOVA. n.s., not significant.

Oxidative stress and increased ROS production are a hallmark of mitochondrial dysfunction in HF. To examine whether IF and PN mitochondria from TAC-operated mice exhibit distinct degrees of oxidative stress compared with control mice, ROS generation was measured in cardiomyocytes using CellROX® Deep Red reagent. Figure 4*a*,*b* shows that cardiomyocytes from TAC-operated mice had significantly higher ROS production in both IF and PN mitochondria, and that increasing pacing frequency to 5 Hz further increased the oxidative stress in both subpopulations of mitochondria. Notably, increase in ROS production in PN mitochondria exceeded that in IF mitochondria, especially at high-frequency pacing (mean ± s.e.m. for IF versus PN mitochondria respectively; 149 ± 19 versus 216 ± 29 a.u., *p* = 0.039).

Compromised mitochondrial Ca^2+^ uptake is a further characteristic of HF. We used saponin-permeabilized isolated cardiomyocytes loaded with Rhod-2 fluorescent indicator and incubated them for 5 min in solutions with increasing [Ca^2+^] to assess mitochondrial Ca^2+^ uptake in IF and PN mitochondria from control and failing mice (figure 4*c–e*). In agreement with previous work [26], we observed slower Ca^2+^ uptake in PN versus IF mitochondria (figure 4*c*,*e*); however, the steady-state amplitude in both subpopulations reached similar levels for each [Ca^2+^] studied. TAC caused a dramatic reduction in mitochondrial Ca^2+^ uptake in both IF and PN mitochondria at each of the three values of [Ca^2+^] studied (figure 4*c*,*d*). Again, functional capability to sequestrate Ca^2+^ was disproportionally impaired in PN mitochondria, which also exhibited a higher threshold level of [Ca^2+^] for uptake compared with IF mitochondria (figure 4*e*; note no uptake by PN mitochondria at 1.35 µM). The seemingly faster Ca^2+^ uptake observed in TAC versus sham PN mitochondria at higher [Ca^2+^] (2 and 10 µM) is likely due to already saturated mitochondrial [Ca^2+^] after exposure to 1.35 µM Ca^2+^ in sham mice.

Ca^2+^ uptake by mitochondria plays an important role in (sub)cellular Ca^2+^ cycling, and its dysregulation has a profound effect on excitation–contraction coupling and cytoplasmic [Ca^2+^] in HF. As PN mitochondria are particularly functionally impaired in TAC animals and failing human hearts, this raises an interesting question as to whether altered Ca^2+^ uptake by PN mitochondria may have a measurable effect on nucleoplasmic Ca^2+^ homeostasis in HF.

### (e) Effect of impaired perinuclear mitochondrial Ca^2+^ uptake on nucleoplasmic Ca^2+^ transients in failing mouse cardiomyocytes

**Figure 5.**
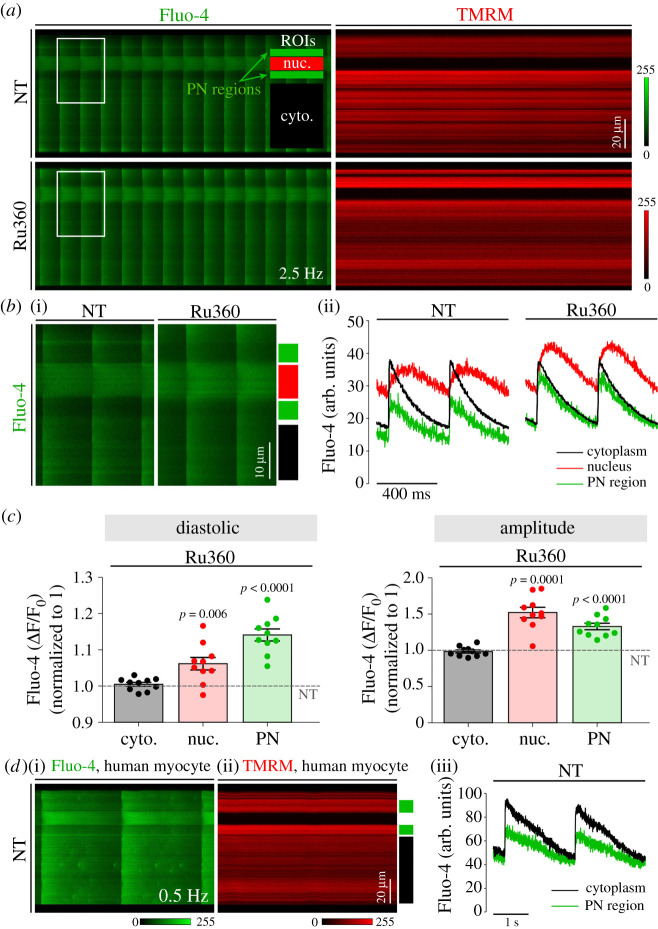
Effect of perinuclear (PN) mitochondria Ca^2+^ uptake inhibition on nucleoplasmic CaTs in healthy murine and human cardiomyocytes. (*a*) Matched line scans of CaTs visualized by Fluo-4 staining (left) and mitochondrial signal visualized by TMRM (red) in healthy murine cardiomyocyte before (NT) and in the presence (bottom) of the mitochondrial Ca^2+^ uptake inhibitor Ru360, stimulated at 2.5 Hz. Regions of interest (ROIs) used for analyses are indicated in the first panel; cytoplasm (black), nucleus (red) and PN regions (green). (*b*) Magnification of corresponding cell areas containing PN mitochondria (i) and matched fluorescence traces of distinct subcellular regions (ii), as indicated in the scheme: cytoplasm (black), nucleus (red) and PN regions (green). (*c*) Average change in Fluo-4 fluorescence signal after the application of Ru360 in cytoplasm (black), nucleus (red) and PN regions (green). The dashed line represents the normalized fluorescence signal before the application of Ru360. *n* = 10 or 11 cells from *N* = 3 mice per group. *p*-values were calculated using one-sample *t*-test for relative difference in fluorescence data. (*d*) Matched line scans of CaTs visualized by Fluo-4 staining (i) and mitochondrial signal visualized by TMRM (ii) in a healthy human cardiomyocyte stimulated at 0.5 Hz (left) and corresponding Fluo-4 fluorescence traces of distinct subcellular regions (iii), as indicated in the scheme: cytoplasm (black) and PN regions (green).

To address this question, we first tested the effect of pharmacological inhibition of mitochondrial Ca^2+^ uptake on nucleoplasmic CaTs in ventricular myocytes isolated from healthy mouse hearts (figure 5*a–c*). Cells were simultaneously labelled with the Ca^2+^ indicator Fluo-4 for quantification of subcellular CaTs and TMRM for detection of mitochondrial localization in the absence and presence of the specific MCU inhibitor Ru360 (10 µM) [29,30]. Ru360 slows mitochondrial Ca^2+^ uptake to the extent that mitochondrial Ca^2+^ uptake on a time scale of action potential-induced CaTs is essentially blocked [31]. In the absence of Ru360, PN regions were clearly noticeable as areas of lower [Ca^2+^] and high TMRM signal surrounding the nucleus on both poles (figure 5*a*,*b*), compatible with the idea that PN mitochondria may buffer Ca^2+^ around the nucleus during electrically stimulated CaTs, therefore shaping the nucleoplasmic-to-cytoplasmic [Ca^2+^] gradients in cardiomyocytes during the cardiac cycle. Similar regions were observed in cardiomyocytes from non-failing human hearts (figure 5*d*), hence strengthening their general applicability and clinical relevance.

The addition of Ru360 had no effect on cytoplasmic CaTs, but dramatically enhanced PN CaTs, as indicated by the significantly higher diastolic Ca^2+^ levels and amplitude (figure 5*b*,*c*). Notably, increased Ca^2+^ cycling in PN regions also translated into higher nucleoplasmic CaTs, in terms of both diastolic [Ca^2+^] and amplitude of CaTs. These data raise the possibility that the lack of PN mitochondrial Ca^2+^ uptake as observed in TAC cardiomyocytes may lead to more rapid propagation of CaTs from the cytoplasm to the nucleus, and moreover, to the disproportional increase in nucleoplasmic versus cytoplasmic CaTs in diseased myocytes, especially at higher pacing rates where mitochondria are expected to take up more Ca^2+^ [32]. Indeed, we previously showed that in TAC cardiomyocytes, higher pacing frequencies elevate diastolic [Ca^2+^] in the nucleoplasm to a much larger extent than in the cytoplasm, leading to the activation of nuclear CaMKII and consequential nuclear export of the transcriptional regulator HDAC4 [33].

**Figure 6.**
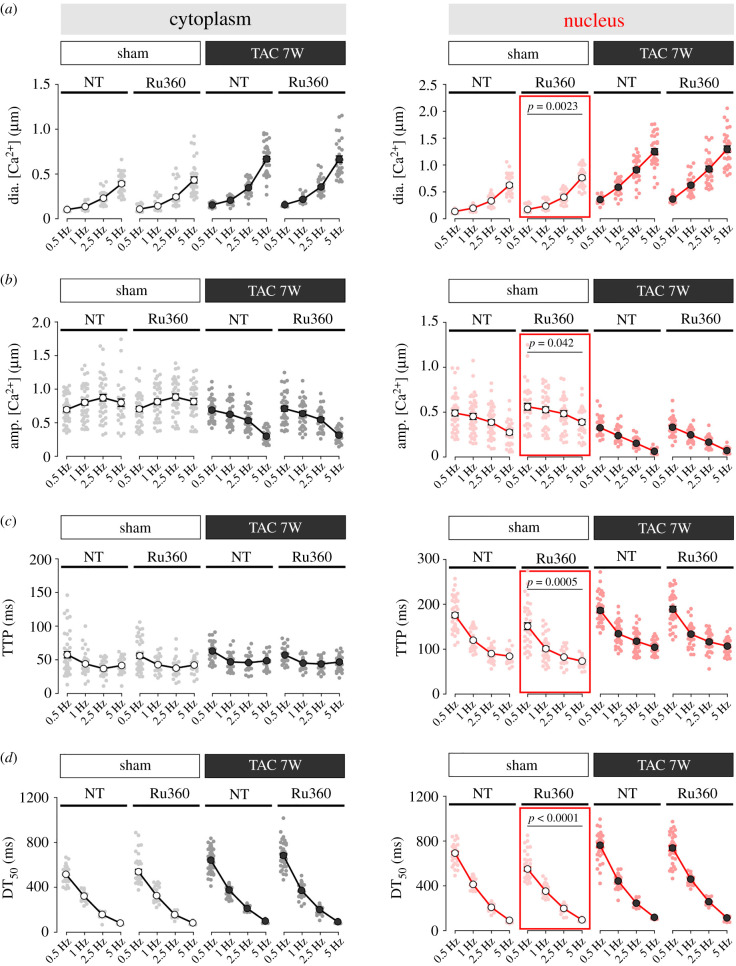
Effect of impaired perinuclear mitochondria Ca^2+^ uptake on nucleoplasmic CaTs in failing mouse cardiomyocytes. Frequency-dependent changes in (*a*) diastolic (dia.) [Ca^2+^], (*b*) amplitude (amp.), (*c*) time to peak (TTP) and (*d*) time from peak [Ca^2+^] to 50% decline (DT_50_) of electrically stimulated CaTs in the cytoplasm (black) versus nucleus (red) of ventricular myocytes isolated from sham (left) and seven-week post-TAC (right) mice, with and without the application of Ru360. *n* = 25–35 myocytes from *N* = 5 or 6 mice per group. *p*-values were calculated using Sidak's *post hoc* test, following significant two-way repeated-measures ANOVA.

Here, we complemented those data with experiments performed in cardiomyocytes from sham- and TAC-operated mice stimulated at increasing pacing frequencies in the absence or presence of inhibition of mitochondrial Ca^2+^ uptake with Ru360 (figure 6). In agreement with our previous work, we show that in TAC-operated mice, diastolic [Ca^2+^] increased, CaTs amplitude decreased and kinetics slowed at any frequency studied. Additionally, changes in nuclear [Ca^2+^] were much more pronounced than those in the cytoplasm. While preincubation with Ru360 caused significant enhancement of nucleoplasmic CaTs in sham-operated control mice (figure 6, red squares), it had no effect on cytosolic [Ca^2+^] in TAC-operated mice. These data are consistent with the finding of blunted PN mitochondrial Ca^2+^ uptake in TAC cardiomyocytes (figure 4*c*,*d*) and suggest that functional impairment of mitochondria can contribute to the disproportionate rise in diastolic [Ca^2+^] in failing cardiomyocytes, especially in the nuclear compartment. Notably, Ru360 had no effect on cytoplasmic [Ca^2+^] in either sham- or TAC-operated mice. The reduction in nuclear CaT amplitude seen in TAC (figure 6*b*, right) is explained by the dramatic rise in diastolic nuclear [Ca^2+^] in failing TAC myocytes (figure 6*a*, right), which limits nuclear Ca^2+^ efflux between beats that is caused by altered Ca^2+^-regulating proteins and nuclear envelope structure [33].

We conclude that the normal buffering of PN [Ca^2+^] by healthy PN mitochondria, which is revealed by Ru360 treatment, is necessary to accelerate nuclear [Ca^2+^] decline, lower diastolic nuclear [Ca^2+^] and lower nuclear CaT amplitudes to limit Ca^2+^-dependent transcriptional signalling. The loss of this Ca^2+^-buffering ability in failing heart PN mitochondria is why Ru360 no longer makes things worse (they are already bad).

## 4. Discussion

The results of the present study indicate that PN mitochondria are functionally altered to a higher degree than IF mitochondria in failing cardiomyocytes, as manifested by the severely depressed Ca^2+^ uptake across a range of different [Ca^2+^], reduced mitochondrial membrane potential and increased ROS production in failing cardiomyocytes exposed to increased frequency of pacing. Depressed Ca^2+^ uptake in PN mitochondria has a direct effect on nucleoplasmic CaTs and may play a causal role in disturbed excitation–transcription coupling and progression of maladaptive cardiac remodelling. These data identify a novel subcellular mechanism of functional decline during HF development that could help the advancement of new cardioprotective strategies and/or better understanding of molecular mechanisms underlying the beneficial effects of current cardiometabolic therapies.

### (a) Differential properties of intrafibrillar and perinuclear mitochondria

Selective mitochondrial autophagy, or mitophagy, removes worn-out and damaged mitochondria with a half-life of approximately 17 days in healthy rat hearts [34], and it is closely linked to mitochondrial biogenesis, which permits cellular replenishment with healthy organelles. By measuring mitochondria and lysosomal co-localization as an index of where mitochondrial turnover is occurring in live cardiomyocytes, Lu *et al*. [26] demonstrated that unfit mitochondria were delivered from intramyofibrillar or SSL regions to the PN area, where they fused with lysosomes to be degraded. If the PN region is the active site for mitochondrial clearance for both IF and PN mitochondria, a concentration of mitochondria with distinct structural and functional properties could be expected in these subcellular spaces in healthy myocytes. In agreement with previous work in rabbit ventricular myocytes [26], we found smaller dimensions, more spherical shape and slower kinetics of mitochondrial [Ca^2+^] rise in PN mitochondria (figures 1 and 4) when compared with IF mitochondria in isolated mouse cardiomyocytes and myocardium. A prolonged (12 day) inhibition of autophagy with 3-methyladenine in cultured neonatal rat cardiomyocytes resulted in preferential accumulation of smaller and less elongated mitochondria especially in the PN regions, suggesting that small mitochondria normally turn over at a higher rate and, therefore, preferentially accumulate following the blockade of autophagy [35]. This may imply that reduction in mitochondrial size and elongation is an important trigger for their trafficking to the PN region and, eventually, the process of recycling.

Impaired mitochondrial removal via mitophagy is a common feature of both compensated myocardial hypertrophy and end-stage HF, suggesting that defective organelle turnover is an early event in cardiac remodelling [21,36]. Under pathological conditions, slower mitochondrial removal would cause their prolonged retention in the IF space, but also potentially lead to a phenotypic shift of IF mitochondria more towards that of a ready-to-be-degraded PN subpopulation. Indeed, our quantitative analysis of ultrastructural mitochondrial morphology and density showed that IF mitochondria from cardiomyocytes isolated from failing hearts appeared more spherical and smaller as compared with controls, making the difference between the two mitochondrial populations less pronounced (figure 1). The longevity of newly formed mitochondria is a key factor to consider in regard to mitochondrial density. Accumulating evidence shows that the absolute abundance of mitochondria is increased in hypertrophic and failing cardiomyocytes, while the autophagy is decreased as early as day 7 post-TAC intervention [37]. This would imply that existing mitochondria have an extended lifespan during cardiac remodelling, a phenomenon also observed in cardiomyocytes from aged rats [34]. Unfortunately, owing to the challenging methodological approach required, direct measurements of the mitochondrial half-life in various organisms and tissue types are scarce, and to our knowledge, no data are available in experimental models of HF or from HF patients.

If true, such extended retention and delayed degradation of IF mitochondria may initially be an adaptive process to compensate for increased energetic demand in hypertrophied cells with myofibrillar volume progressively expanding in response to mechanical overload [23]. In conditions of fast myofibrillar growth, slowing the mitophagy process may prevent depletion of the mitochondrial pool, which if it falls below the required level for cardiac contractile activity or maintenance of cellular integrity will lead to deterioration in cardiac function and eventually to the death of individual cardiomyocytes [38]. In support of this possible compensatory scenario, we found that the mean relative cross-sectional area populated by both IF and PN mitochondria was reduced in hypertrophic and failing cells when compared with controls (figure 2), despite the increase in number of organelles per cellular area and absolute mitochondrial content. When proliferation of new mitochondria is not enough to prevent their dilution by the growing myofibrillar elements, their slower degradation may help, at least in the short-term, by alleviating the energetic deficit of beating cardiomyocytes.

### (b) Reduced mitochondrial function in heart failure, especially perinuclear Ca^2+^ uptake

IF mitochondria retained beyond their regular turnover rate would be expected to show slightly decreased functional properties and be especially vulnerable to stress in hypertrophic and failing cardiomyocytes. In addition, those sent to be recycled in PN spaces would show more pronounced signs of functional exhaustion, with potential consequences to overall cardiomyocyte vulnerability including functional and structural outcomes. Pressure overload-induced HF has been shown to result in increased mitochondrial oxidative stress and ROS generation and reduced Δ*Ψ*_m_, Ca^2+^ uptake and ATP production [21,39], observations that are consistent with HF development in the current study. More specifically, our recent data from the same animal model [21] demonstrated that ATP generation and total mitochondrial reserve capacity were severely reduced in TAC versus sham cardiomyocytes. We monitored mitochondrial redox potential by FAD/FADH_2_ autofluorescence under conditions of increased cell work induced by high-frequency pacing. Quantitative analysis in TAC cardiomyocytes revealed nearly maximal FAD/FADH_2_ ratio (oxidized) at basal conditions, and the TAC cardiomyocytes were significantly more oxidized than sham cardiomyocytes. In addition, it has recently been demonstrated that pressure overload induced by TAC resulted in a significant increase in the expression of Nox2 oxidase subunits and a more oxidized state overall as assessed by the GSH/GSSG ratio [40]. It is abundantly clear that cardiac mitochondrial ROS emission is dynamically regulated by Na^+^, Ca^2+^ and the mitochondrial redox environment, a concept comprehensively elaborated upon by Cortassa *et al*. [41]. This concept postulates that healthy cardiac mitochondria are fine-tuned to an intermediate redox state that prevents excessive ROS generation under highly reduced conditions, while also maintaining an anti-oxidative capacity under highly oxidized conditions. Our data support this concept, as the intermediate basal FAD/FADH_2_ ratio with minimal ROS generation in sham cardiomyocytes shifted to depressed Ca^2+^ uptake, and severely oxidized FAD/FADH_2_, with a concordant increase in ROS generation in TAC mice.

The most novel finding here is that, while IF mitochondria showed signs of exhaustion mostly under stress, PN mitochondria from failing myocytes showed signs of functional deterioration already at baseline and high frequency of pacing pushed them into even more oxidative stress and functional decline (figures 3 and 4). This is in agreement with our working hypothesis that in HF, mitochondria are over-worked before they undergo the process of recycling and that PN regions contain a population of especially vulnerable mitochondria which are at the terminal stage of their life cycle. Excessive ROS generation by exhausted mitochondria may damage proteins, lipids and mitochondrial DNA, leading to further loss in their bioenergetic capacity, fusion and fission disbalance, and decreased mitophagy. Over time, this leads to the accumulation of depolarized and ROS-hyperproducing mitochondria, contributing to the development of cardiovascular diseases [42]. Taken together, although potentially beneficial against energetic deficit in the initial phase of hypertrophy, slowing the removal of IF mitochondria could cause excessive ROS generation by unfit organelles. Finally, as mitochondria become more damaged and more ROS are generated, they may induce cell death and functional decay in an enlarged myocardium [43].

Another important parameter of mitochondrial fitness with implications in a variety of cellular functions is their ability to take up Ca^2+^, especially at higher cytoplasmic Ca^2+^ levels. In HF, diminished SR Ca^2+^ release and increased intracellular Na^+^ levels depress mitochondrial Ca^2+^ uptake, which impairs their capacity for sustaining optimal matrix NAD(P)H redox potential. This in turn boosts oxidative stress, especially upon increased workload. Here, we found severely diminished IF mitochondrial Ca^2+^ uptake in failing cardiomyocytes, and notably, even more depressed Ca^2+^ uptake by PN mitochondria (figure 4). Although enhancing mitochondrial Ca^2+^ uptake could potentially increase the chances of Ca^2+^ overload and opening of the mitochondrial permeability transition pore as described in conditions of acute ischaemia/reperfusion [44], recent studies described a plethora of beneficial effects it has on cardiomyocyte homeostasis under physiological and pathophysiological conditions. For example, work by Liu *et al*. [24] showed that moderate overexpression of MCU in a guinea pig model of pressure overload-induced HF inhibited mitochondrial oxidative stress, enhanced contractility and responses to β-adrenergic stimulation, and inhibited arrhythmias. Along the same lines, preventing the TAC-induced reduction in mitochondrial Ca^2+^ uptake by cardiac-specific deletion of the translocator protein of the outer mitochondrial membrane (TSPO) protected mice from developing a full-blown HF phenotype [21]. In addition to genetic manipulations, pharmacological approaches to inhibit or stimulate mitochondrial Ca^2+^ uptake had clear whole-cell functional outcomes in isolated rabbit atrial myocytes [45]. Specifically, exposure of cardiomyocytes to Ru360 propelled the occurrence of pacing-induced CaT alternans, while the opposite was true for pharmacological stimulation of mitochondrial Ca^2+^ uptake with the polyamine compound spermine.

### (c) Perinuclear Ca^2+^ buffering by perinuclear mitochondria limits nuclear Ca^2+^ signalling

One unexplored mechanism for the efficacy of therapeutic approaches targeted to increase mitochondrial Ca^2+^ uptake in preventing cardiac remodelling and its transition to HF could be their ability to specifically influence (peri)nuclear Ca^2+^. This idea is supported by the prominent mitochondrial accumulation in PN regions and the space between the two nuclei in characteristically binucleated ventricular cells (figure 1). To explore the possibility that the lack of mitochondrial Ca^2+^ uptake in the PN regions could result in the fast and robust propagation of a Ca^2+^ signal toward the nucleus, we blocked MCU via acute Ru360 application. In the presence of Ru360, cytoplasmic CaTs remained fast and unaltered, suggesting that even if there is some mitochondrial Ca^2+^ uptake on a beat-to-beat basis, it is too low to substantially alter global cytosolic CaTs. However, propagation through the PN region was significantly enhanced when mitochondrial Ca^2+^ uptake was blocked (figure 5). Furthermore, in the presence of Ru360, nucleoplasmic CaTs were larger, and slower to decline, resulting in elevated diastolic [Ca^2+^] levels, effects consistently observed over a range of increasing pacing frequencies (figure 6). The data suggest that rapidly propagating cytoplasmic CaTs are not appreciably affected by mitochondrial Ca^2+^ sequestration, but that nucleoplasmic CaTs are significantly shaped by the Ca^2+^ buffering effect of PN mitochondria. This normal ability of PN mitochondria to protect the nucleus from pacing-induced Ca^2+^ loading is lost in failing myocytes, and that loss may contribute significantly to elevated nuclear Ca^2+^ loading and signalling to nuclear transcription. Furthermore, our data are in agreement with a previous study in atrial myocytes which found that centrally located mitochondria can modulate Ca^2+^ wave propagation in the central region of isolated atrial myocytes [31].

The increase in (peri)nuclear Ca^2+^ levels due to the reduction in mitochondrial Ca^2+^ uptake especially around the nucleus may promote activation of Ca^2+^-mediated hypertrophic signalling and gene transcription. Indeed, our recent work showed that the PN region is a fine-tuned microdomain for local Ca^2+^-mediated transcriptional regulation [46]. For example, enhanced nucleoplasmic Ca^2+^ levels may activate nuclear Ca^2+^-calmodulin-dependent protein kinase II (CaMKII), which can phosphorylate histone deacetylase 4 (HDAC4) and drive its nuclear export. This process mediates the de-repression of Mef2-dependent transcription, implicated in the development of HF [47]. Increased PN Ca^2+^ levels may, on the other hand, ensure a pool of active PN CaMKII which will keep rephosphorylating any exported HDAC4 that becomes dephosphorylated after leaving the nucleus. This could prevent HDAC4 from reentering the nucleus and reinforce the transcriptional signalling of activated CaMKII.

One important aspect to consider when interpreting the present data is the variety of HF phenotypes in regard to left ventricular ejection fraction, pathophysiology, underlying triggers (e.g. myocardial infarction, hypertension and toxic agents), mechanisms of progression and response to treatment. The development of powerful techniques for comparative transcriptome analysis of cardiomyocytes and non-myocytes on a single cell level identified numerous aetiology-specific alterations in gene expression, pointing to different underlying mechanisms of the disease progression [48]. This highlights the importance of careful interpretation of data from a particular experimental animal model, and the need to exercise caution when embedding results in the context of human disease. For example, rapid introduction of strong stressors such as TAC in young animals tends to lead to an HF phenotype with reduced ejection fraction. While beyond our present scope, observations documented here could also be involved in mediating cardiac remodelling in HF subtypes that differ in terms of aetiology. For example, it would be interesting to see if similar alterations in cytoplasmic/mitochondrial/nucleoplasmic Ca^2+^ crosstalk occur in the slower disease progression observed in animal models and patients who suffer from HF with preserved ejection fraction.

Taken together, the present study implicates fitness and functionality of PN mitochondria as an important determinant of cardiac remodelling that may—via shaping nucleoplasmic Ca^2+^ levels—contribute to the development and progression of hypertrophy and HF. Normalization of mitochondrial Ca^2+^ regulation may therefore be a novel therapeutic approach to restore altered Ca^2+^-mediated transcription and prevent adverse cardiac remodelling.

## Data Availability

The data supporting findings of this study are available from the Dryad Digital Repository: https://doi.org/10.5061/dryad.pvmcvdnn9 [49]. Materials and methods are described in detail in the electronic supplementary material [50].
